# Self-supply groundwater in five communities: Moshie Zongo, Aboabo, Kotei, Ayeduase and Apemso in Kumasi Metropolis, Ghana

**DOI:** 10.1016/j.heliyon.2023.e23823

**Published:** 2023-12-19

**Authors:** Noel Bakobie, Helen M.K. Essandoh, Sampson Oduro-Kwarteng, Emmanuel Kwame Appiah-Adjei, Shaikh Ziauddin Ahammad, Sumedha Chakma

**Affiliations:** a Regional Water and Environmental Sanitation Centre, Kumasi (RWESCK), Department of Civil Engineering, Kwame Nkrumah University of Science & Technology, Kumasi, Ghana; b Department of Environment, Water and Waste Engineering, School of Engineering, University for Development Studies, Nyankpala-Tamale, Ghana; c Department of Civil Engineering, College of Engineering, KNUST, PMB, Kumasi, Ghana; d Department of Geological Engineering, College of Engineering, KNUST, PMB, Kumasi, Ghana; e Department of Biochemical Engineering & Biotechnology, Indian Institute of Technology, Delhi, India; f Department of Civil Engineering, Indian Institute of Technology, Delhi, New Delhi, India

**Keywords:** Alternative water, Boreholes, Drinking water, Survey, Water quality

## Abstract

Self-supply water has been acknowledged as a viable alternative to meeting the water needs of inhabitants. This study was designed to determine the main issues that influence self-supply water coverage in five (5) communities in the Kumasi Metropolis. The research employed a well-structured questionnaires and a total of 369 households were surveyed. The Statistical Package for Social Sciences (SPSS) version 26 and Microsoft Excel (2016) tools were used to analyse the data. The outcomes of the research show that a greater number of the respondents (77 %) did not have connections to the Ghana Water Company Limited (GWCL) distribution system. Approximately, 69 % of respondents had access to alternative water sources with mechanized boreholes forming the majority (32 %). However, a greater number of the respondents (64 %) did not disinfect their water to make it potable. The most favourable drinking water source for a greater number of the residents (51 %) was sachet water. The study showed there was a significant association between respondents' type or source of water with religion (p < 0.000), household size (p < 0.000), duration of stay (p = 0.026) and number of dependents (p = 0.006). However, there was no association between type or source of water with educational level (p = 0.130), occupation (p = 0.310), income level (p = 0.139) and type of home (p = 0.102). This study revealed that self-supply is contributing to the water needs of some residents in Kumasi and could contribute to the country's accomplishment of SDG 6.1 if residents ensure that it is safely managed. To broaden the scope of the study and the impact of self-supply groundwater, additional studies should be conducted in other communities, as well as the extent of other beneficiaries who have access to self-supply facilities other than the owners.

## 1 Introduction

Self-supply is an on-site water supply system operated by a household, and this type of supply uses groundwater or rainwater [1]. In urban areas, self-supply of water refers to households or families developing their own water sources rather than relying on the public water supply system [2,3]. Self-supply facilities are hand-dug wells constructed to access shallow groundwater, deep wells that can access both shallow and deep groundwater, and other sources like springs and rainwater harvesting [4].

Studies have shown that the use of self-supply groundwater in urban areas of developing countries is widespread and a component of the total water supply [[5], [6], [7], [8], [9]]. For example, it is estimated that approximately 125 million people in Sub-Saharan Africa and 340 million people in India rely on self-supply [10]. Self-supply accounts for approximately 35 % of total water supply to Sao Paulo in Brazil, and 30 % of Lagos residents obtained their water from self-supply in 2009 [10]. In the EU and USA, “groundwater provides the public water supply for 310 and 105 million people, respectively” [10].

However, authorities largely disregard this effort, and very little information is available in most developing countries about the contribution of self-supply groundwater to residents' water needs. Humanity requires access to safe drinking water and sanitation services in order to maintain proper health and welfare. Potable drinking water is a fundamental human right; however, access to it is a worldwide concern [11]. According to the UN Joint Monitoring Programme (JMP [12], nearly 2 billion people do not have access to potable, dependable, cheap, and easily available drinking water supplies. As at 2019, 3.6 billion (47 %) of the population of the world live in areas with limited water supply [13].

Researchers have shown that in many cities around the world, water demand exceeds supply necessitating self-supply for a variety of reasons. Some of the reasons include those beyond the reach of utilities [14,15], those served by municipal water systems, and those with insufficient water supplies [14]. Other reasons include rising population growth due to user demand and industrialization [15], increased awareness and low-cost technology, and a desire to source water locally [7], and as a long-term cost-cutting strategy [9,16].

In Ghana, there are major challenges with access to drinking water just as in other parts of the developing world. Despite these challenges, there has been progress in the water sector, with 79 % of the entire people in Ghana having access to basic drinking water, with 93 % of this percentage in the urban areas and 68 % in the rural areas [17]. According to the Ghana Living Standard Survey (GLSS) [18], 27.3 % of Ghanaian households access their drinking water from piped-borne sources, 28.5 % from wells (boreholes and hand-dug wells) whilst the remaining 44.1 % utilize other sources (bottled water, rainwater, etc.). Access to safe drinking water services increased by 28 % in Ghana, from 13 % in 2000 to 41 % in 2020 [19]. Coverage of basic drinking water services increased by 22 % during the same period, from 64 % in 2000 to 86 % in 2020 [19]. From the year 2000, Ghana access to safely managed drinking water has been the fastest in Sub-Saharan Africa increasing by 1.4 % yearly to 41 % by 2020 [19]. This rate is not enough if Ghana is to achieve universal access by 2030 [19]. The projected population of Ghana by 2025 will be 35 million with a sharp rise in demand for water and sanitation services as 63 % of that population will be living in urban areas [20]. The strategy of the Ministry to achieve universal coverage for water services is to increase the national water coverage rate from 80 % in 2015 to 100 % in 2025 and urban water coverage from 85 % in 2015 to 100 % in 2025 [20].

The Government of Ghana, Development Partners, and NGOs are the primary sources of funding for the WASH sector, but current levels of WASH financing are insufficient to meet WASH Sustainable Development Goal (SDG) targets [21]. It is estimated that more than GHS 6.0 billion per year is required to meet the SDG 6 targets for water and sanitation. However, total public funding in 2021 was 10.13039/100001126GHS 560 million, accounting for less than 10 % of total financing requirements [21]. It is abundantly clear that the state cannot provide consistent water supply to residents in the majority of developing countries [7]. The situation is exacerbated by rapid population growth and residents living in emerging areas where it is difficult to reach out to such residents.

In order to ensure stable and sufficient household water supply, most residents in Ghana rely on various water sources [22]. Thus, self-supply of water in emerging and municipal areas in Ghana is becoming a widespread phenomenon as water supply from the piped systems of the Ghana Water Company Limited is interrupted or non-existent in some places. In Ghana, the proportion of municipal residents who have piped water has fallen from 41 % in 1990 to 32 % in 2015, forcing peri-urban residents to rely on shallow groundwater sources to meet their water needs [23]. Concerning access to safe drinking water, the situation in Kumasi, the capital city of the Ashanti Region, is similar to that in other parts of the country [24]. In the Ashanti Region of Ghana, 53.6 % of residents’ access piped-borne water for domestic purposes. On the other hand, 41.6 % of the residents use well water whilst 4.8 % use alternative sources [18].

The Ghana Water Company Limited (GWCL) supply gap in Kumasi can be attributed to a variety of factors. Financial constraints, non-expansion of existing water treatment facilities to meet growing water demand, availability of water resources, growing urban population, development of new areas, and the inability to use groundwater for water supply due to quantity challenges are some of the possible reasons for the supply gap and areas not covered by GWCL [25]. Most residents in emerging and municipal areas rely on self-supply of groundwater to fill the water supply gap [7]. This practice of self-supply is widespread in Kumasi's peri-urban and urban areas.

Self-supply water could play a significant role in Ghana accomplishing the Sustainable Development Goals (SDGs) 6.1 because it provides water into homes, which is a measure for water service to be recognised as safely managed. Despite the enormous benefit self-supply of water, it has not been well recognised by authorities as means of improving the peri-urban and urban water supply shortfalls.

The majority of research in Kumasi has concentrated on the drinking water quality of self-supply groundwater. For example [26], assessed both physico-chemical and microbial water quality in nine (9) communities in Kumasi [27]; in hostels around five communities (Ayeduase, Bomso, Kentintikrono, Kotei, and New Site); and [28,29] in Kotei and Ayeduase respectively. On the other hand [30], assessed the physico-chemical and microbial quality in the environments of Kumasi. On only microbial water quality of self-supply groundwater [31], conducted studies in four communities (Aboabo, Ayigya, Kentinkrono, and Gyinyase) [32], in three communities (Aprade, Domeabra, and Mesewam) [33], in three communities (Ayeduase, Kotei, and Boadi), and [34] in Moshie Zongo [35]. investigated heavy metal contamination in groundwater near the Oti landfill in Kumasi, whilst [24] evaluated the water quality of domestic drinking water in Kumasi's Oforikrom Municipality. In all these studies, no research was carried out on the extent and factors that necessitate self-supply groundwater. In terms of available information, very few research works have been carried out in Kumasi, particularly on the extent and factors that necessitate self-supply groundwater though some studies have been conducted in other parts of the country [7,36].

The key objectives of this study were to: (1) ascertain the degree of self-supply groundwater coverage in five (5) communities in the Kumasi Metropolis; (2) examine the association between household characteristics and preferred source of self-supply; (3) find out the reasons that necessitate self-supply in the communities; (4) ascertain residents' perceptions of the quality of self-supply water; (5) highlight self-supply water initiatives. (6) assist in the formulation of policies for better groundwater resource development and exploitation.

We make two contributions to knowledge. First, this study adds to the scant literature on the extent of self-supply groundwater by residents in peri-urban and urban areas in Ghana. Second, the findings of this paper are expected to assist policymakers in understanding the existing challenges to self-supply in urban and peri-urban areas, as well as in implementing measures to mitigate negative health effects.

## 2 Materials and methods

### 2.1 The study area

**Fig. 1:**
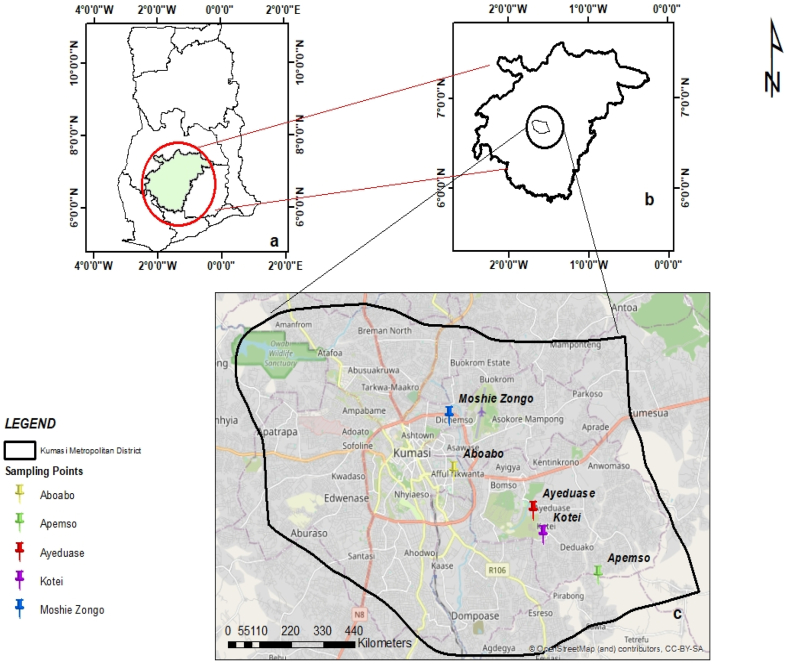
Location of **(a)** Ashanti Region in Ghana, and **(b)** Kumasi Metropolis with **(c)** study communities.

Source: By Authors

The research was conducted in five (5) communities in the Kumasi Metropolis. The communities are Moshie Zongo, Aboabo, Ayeduase, Kotei and Apemso (see Fig. 1).

These communities were selected because of their dependence on groundwater supplies.

Kumasi is Ghana's second largest city, located approximately 270 km north of the country's capital, Accra, between latitudes 6°35 and 6°4 N and longitudes 1°30 and 1°35 E. The city is located in a humid, semi-arid climate zone with a weakly bimodal rainfall pattern that peaks in June and September [37]. The average annual rainfall is 1448 mm and the average annual temperature is 28 °C whilst the monthly temperatures vary between 21.5 and 30.7 °C. The four major rivers that flow through the city and drain into the Oda River are the Sisa, Wiwi, Daban, and Subin [38]. These surface water bodies also serve as domestic water sources and are predominantly used for laundry and non-direct consumption purposes.

### 2.2 Study design and approach

**Fig. 2:**
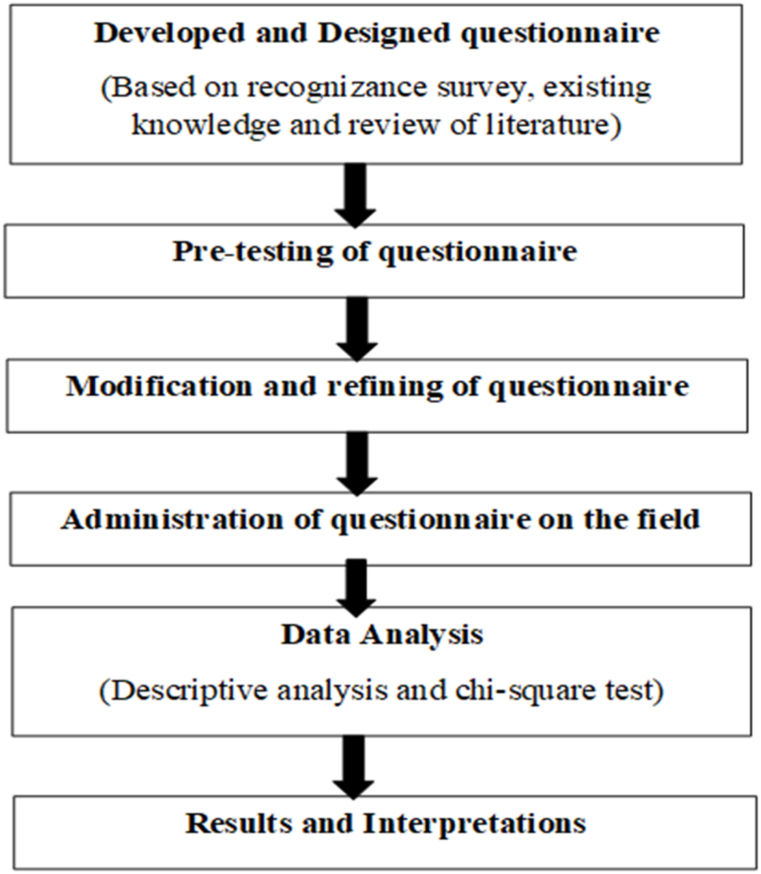
Flow chart for questionnaire process in the study area.

A cross-sectional research design was the most appropriate type of research design for this study because it was less expensive and time-consuming than other known research designs [39]. It also allowed the researchers to gather data from a large pool of participants and compared the differences and similarities between them. Fig. 2 is the flow-chart for the questionnaire process for the study areas.

The Cochran method (1963) [40] was used to calculate the sample size because we did not have the population of the study communities.

No = Z^2^pq = 1.9^2^ (0.5) (0.5) = 384 participants

E^2^ 0.05.^2^

Where No = sample size, Z = standard Normal value base on confidence level, p = sample proportion, and e = error percentage.

The target sample size was 384 participants based on the sample calculation method described above. Based on study site scouting, Kotei, Ayeduase, and Aboabo had similar water supply characteristics (roughly the same proportions of protected and unprotected water supply systems), whereas Apemso and Moshie Zongo had, high protected and high unprotected self-supply sources, respectively. As a result, the 384-sample size was distributed as follows: Kotei (60), Ayeduase (60), Aboabo (60), Apemso (100), and Moshie Zongo (104). This was done to ensure that the communities produced balanced results.

The study employed a well-structured questionnaire that was divided into two categories (A and B). Category A captured the demographic characteristics of respondents whilst category B considered households’ water coverage and sources and respondents' perceptions of water supply issues [24,36]. The questionnaires were pre-tested on 30 participants in a non-study location and revised before they were administered. Pre-test sample was not included in the analysis. Five (5) communities were purposively selected based on access to or non-access to the water supply network of Ghana Water Company Limited. These communities were largely characterized by the high number of self-supply compounds/households based on scouting in the study areas. In addition, eligible compounds/households were those that have self-supply groundwater.

To administer the research tools, three research assistants were trained and hired. A total of 369 complete responses were obtained from randomly visited compounds in the study communities, resulting in a 96 % response rate with a 4 % response error. The compounds/households for the data collection were selected based on the existing sections of the areas in the communities in order to have a representative data. The compounds/households were then randomly selected to give equal chances and then the questionnaire administered if the compound/household uses self-supply. Targeted respondents were household heads, but in their absence, any household member aged 21 and above with good knowledge of the household characteristics, including water supply and related issues, was targeted as a respondent. This selection criterion was designed to ensure that respondents understood the purpose of the survey and were willing to participate in it by signing a consent form.

The Committee on Human Research Publication and Ethics (CHRPE) of Kwame Nkrumah University of Science and Technology approved the study and the associated documents such as the consent form and questionnaire with the approval number (CHRPE/AP/546/22).

The Statistical Package for Social Sciences (SPSS) version 26.0 and Microsoft excel office 2016 computer software programmes were used to analysed the data. Data collected were quantitative in nature; descriptive analysis was used to present the socio-demographic characteristic, water supply ownership, and perception of water quality and treatment methods of respondents. The data was analysed using cross-tabulation and Pearson Chi-square tests of association (at the 5 % level of significance) and descriptive statistics were used to test the hypothesis on some variables of interest.

## 3 Results and discussion

### 3.1 Demographic features of respondents

The demographic features of the interviewees included sex, age, marital status, educational level, occupation, monthly income, religion, household size, number of dependents, duration of stay, and type of home and p-values of the chq-square analysis are summarised in Table 1.

**Table 1:** Demographic features of respondents & p-values from chi-square analysis.

	Frequency	Percent	p-value
**Sex (N** = **356)**			0.719
Male	125	35.11	
Female	231	64.89	
**Age (N** = **358)**			0.706
Less than 26 years	70	19.55	
26–35 years	105	29.33	
36–45 years	88	24.58	
46–55 years	41	11.45	
56–65 years	31	8.66	
66 and above	23	6.42	
**Marital status (N** = **359)**			0.484
Married	231	64.35	
Single	90	25.07	
Widow/widower	27	7.52	
Divorced	5	1.39	
Separated	6	1.67	
**Educational level (N** = **359)**			0.130
Primary	69	19.22	
JHS	78	21.73	
Secondary	63	17.55	
Post-Secondary	33	9.19	
Tertiary	87	24.23	
None	28	7.80	
Other	1	0.28	
**Occupation (N** = **356)**			0.310
Self-employed	116	32.58	
Trader	108	30.34	
Government employee	32	8.99	
Private sector	17	4.78	
NGO	5	1.40	
Other	78	21.91	
**Monthly Income (N** = **301)**			0.139
< GHC 500	131	43.52	
500–1000	122	40.53	
1001–2000	43	14.29	
2001–3000	5	1.66	
**Religion (N** = **358)**			< **0.001**
Christian	219	61.17	
Muslim	127	35.47	
Traditional	12	3.35	
**Household size (N** = **352)**			< **0.001**
<5 persons	126	35.80	
5–9	151	42.90	
10–14	39	11.08	
15–20	15	4.26	
>20	21	5.97	
**Number of dependents (N** = **336)**			**0.026**
<3 persons	143	42.56	
3 - 6	152	45.24	
7 - 10	31	9.22	
11+	10	2.98	
**Duration of stay (N** = **342)**			**0.006**
<5 years	99	28.95	
5 - 9	101	29.53	
10 - 14	58	16.96	
14 - 20	25	7.31	
>20	59	17.25	
**Type of home (N** = **357)**			**0.102**
Private	143	40.06	
Compound house	214	59.94	

The research showed that a greater number of the interviewees 231 (64.89 %) were females whilst 125 (35.11 %) were males as shown in Table 1. At the 5 % level of significance, there was no significant relationship between sex and water source type (p = 0.719) (Table 1). According to the chi-square analysis, females (62.70 %) contributed more to this insignificance than men (37.30 %) (see Supplementary sheet). The majority of females in the study could be attributed to the fact that they manage households’ water supply as corroborated by Appiah-Effah [24] when they conducted similar research in the Oforikrom Municipality (Kumasi, Ghana) and [41] in Vietnam. However, this findings is contrary to a study in Pakistan where the majority of the respondents (88 %) household heads were males [42] and in India 99.5 % [43].

Furthermore, the respondents' ages ranged from less than 26 years to over 66 years. Those less than 26 years were 70 (19.55 %), 193 (53.91 %) were within the age bracket of 26–45 years, 72 (20.11 %) belong to the age bracket of 46–65 years and the remaining 23 (11.45 %) were over 66 years (Table 1). At the 5 % level of significance, there is no significant relationship between respondents' age and the type of water used (p = 0.706) (see Supplementary sheet). The majority of respondents 193 (53.91 %) are between the ages of 26 and 45, indicating that the population is young. This finding corroborates a study by Abanyie [36] in Doba and Nayagenia, Ghana, where there was youthful dominance in the number of respondents.

In addition, most of the participants 231 (64.35 %) were married, 90 (25.07 %) were reported to be single, 27 (7.52 %) were widowed, 5 (1.39 %) were reported to be divorced and 6 (1.67 %) said they were separated as at the time of the study (Table 1). There was no association between marital status and the source of water of the respondents (p = 0.484) (Table 1). The biggest contributor to the non-association was from married couples (65.74 %) from the chi-square analysis (see Supplementary sheet). Thus, irrespective of the marital status what people care more about was their water need. Not only that, but also this research revealed that 210 (58.50 %) of the interviewees had completed basic education and secondary education, 33 (9.19 %) had completed post-secondary education (nursing, teacher training, polytechnic) 87 (24.23 %) had completed their tertiary education and 28 (8.08 %) were not educated (Table 1). The study revealed that a greater number of the interviewees (92 %) had received some form of formal education. However, there is insignificant association at the 5 % level of significance between the educational level of the respondents and the type or source of water used in the study area (p = 0.130) (Table 1). Tertiary educational level accounted for 25.20 % of the non-association (see Supplementary sheet). Clearly, education had no influence on the respondents' choice of water type or source because water is life, and getting water was the bottom line for majority of them. But contrary to our findings, a study in Pakistan found that educated respondents were more particular about the choice of their water sources [42].

Likewise, majority of the participants 302 (84.83 %) reported that they were self-employed, 32 (8.99 %) were Government employees and 22 (6.18 %) were private sector workers (Table 1). The research showed that a greater number of respondents worked for themselves (85.00 %) but there was no significant association between occupation and the type or source of water by the participants (p = 0.310). Chi square analysis revealed that the biggest contribution to the non-association was from self-employment (82.40 %) (see Supplementary sheet). This implies that their primary goal, regardless of occupation, was to obtain water for their domestic needs. Furthermore, a greater number of the respondents 131 (43.52 %) reported that they earned less < GHS 500, 122 (40.53 %) earned between 500 and 1000 and the rest 48 (15.95 %) earned between 1001 and 3000. A greater number of the interviewees had an income of less than GHS 500.00 (<$50) per month. However, no significant relationship between respondents' monthly income and the type of water source was observed (p = 0.139). This suggests that the income level of residents did not influence the choice of their water source but they rather saw water as a necessity. These findings corroborates the works of [15] who stated that there was no differences in the choice of water type and the income level of residents. In addition, as stated by Ref. [8], for the poorest self-financed water supply is a necessity whilst for the richest it offers greater autonomy and assurance of a reliable supply. However, our findings contradict those of other researchers who discovered that affluent people or well-organized communities were more likely to consider self-supply groundwater [6,9,42,44] and those who utilize water for commercial purposes.

From the study 219 (61.17 %) were Christians, 127 (35.47 %) were from the Islamic religious background, and 12 (3.35 %) were traditionalists (Table 1). In comparison to other religions or beliefs, majority of the interviewees in the study were Christians. The religion of respondents was significantly associated with the type or source of water at 5 % error level (p < 0.001). Christians accounted for about 63.05 % of the statistical association between religion and the source of water while Muslims accounted for 32.13 % (see Supplementary sheet). In addition, Christians were more likely to use mechanized boreholes (24.50 %) and boreholes (21.69 %) compared to Muslims 5.22 % and 7.23 % respectively. Muslims on the other hand were found to use protected hand dug wells (14.06 %) compared to Christians (8.84 %) Table 1.

In terms of household size, 126 (35.80 %) of respondents had a household size of 5, 151 (42.90 %) had a household size of 5–9, 39 (11.08 %) had a household size of 10–14, 15 (4.26 %) had a household size of 15–20, and 21 (5.97 %) had a household size of >20 (Table 1). There was significant association between household size of the respondents and the type/source of water (p < 0.000). The household size of 5–9 accounted for 42.68 % of the association followed by household size of less than 5 (37.40 %) (see Supplementary sheet). This suggest that smaller household sizes, probably in private homes were more likely to have water systems in their homes as compared to larger family sizes who were most likely to be in compound houses. This observation is contrary to the findings of [42] in Pakistan where large household sizes were more likely to have water systems at home. Furthermore, the majority of respondents 152 (45.24 %) had dependents ranging from 3 to 6 people, 143 (42.56 %) had dependents less than 3 people, 31 (9.22 %) had dependents ranging from 7 to 10 people, and 10 (2.98 %) had dependents of 11 or more people. The number of dependents was statistically significant with the type/source of water of the respondents (p = 0.026). Household with dependents of three to six (3–6) accounted for most of association (46.58 %) followed by households with members less than three (<3) (41.45 %) (see Supplementary sheet).

When asked how long they had lived in the vicinity, a greater proportion of respondents 101 (29.53 %) had stayed for 5–9 years, 99 (28.95 %) for less than 5 years, 59 (17.25 %) for more than 20 years, 58 (16.96 %) for 10–14 years, and 25 (7.31 %) for 15–20 years (Table 1). The study discovered that the type or source of water was statistically associated with the duration of stay in a specific area (p = 006) (Table 1). The study revealed that new settlers (less than five years) accounted for the greatest percentage of association (34.17 %) of the various types/sources of water followed by those who stayed for five to nine (5–9) years (see Supplementary sheet). For those who stayed for less than five years, they were more likely to own boreholes with hand pumps (11.67 %) while those who stay from five to nine years mostly owned mechanized boreholes (12.08 %) (see Supplementary sheet). This finding could be because of the inability of residents to get access to water supply from GWCL prompting them to make provision for themselves. This observation could influence proper planning in these localities and can contribute to water contamination. In terms of home type, as shown in Table 1, 206 (59.94 %) lived in compound houses and 143 (40.06 %) lived in private houses. According to the survey, more than half of respondents lived in a compound house. Statistically, there was no association which was significant between the type of home and the type of water source (p = 0.102). However [22], discovered that large homes or households would prefer to vary their water sources.

### 3.2 Provision of water by the owner

According to the survey, approximately 84 (23.40 %) of respondents had access to the Ghana Water Company Limited's water supply system, while the remaining 275 (76.60 %) did not (Table 2). The chi-square analysis revealed that there was significant association between the connection of respondents to GWCL pipe system and the type of water source (p = 0.024) at the 5 % level of significance. The major contributor to this significance was respondents who are connected to the GWCL piped system (86.00 %) as compared to those who are connected (14.00 %) (see Supplementary sheet). The outcomes of our research show that majority of the interviewees were not connected to the Ghana Water Company Limited (GWCL) distribution system. This finding was not surprising given that the majority of the residents in the study areas were located outside of the city centre, with the exception of Moshie Zongo and Aboabo, which are served by the GWCL supply systems. Even in these areas, some residents chose not to be connected to the supply systems and instead sought their own water supply. However, this observation is contrary to the findings of [45] who found that in Kumasi, about 95 % of residents could access piped water in their premises or at nearby standpipes. Probably, this could be the result of rapid urbanisation in Kumasi making it difficult for the utility services to meet the water needs of inhabitants and this could account for the disparity. In contrast to our findings [7], discovered that approximately 57 % of residents in Dodowa, Greater Accra, had access to GWCL water supply as their primary source, with approximately 55 % having a secondary source as a backup. In addition, a recent study during the COVID-19 pandemic in Ghana revealed that 58.70 % of residents relied on GWCL supply systems as their main source of water, 33.10 % were self-supplied and 8.00 % relied on Community Water and Sanitation Agency (CWSA) for water supply [46].

**Table 2:** Owner provision of water & p-values from chi square analysis.

Question	Frequency	Percent (%)	p-value
Connected to Ghana Water Company Limited water supply			0.024
Yes	84	23.4	
No	275	76.6	
**Other source of water in this house**			**0.138**
Yes	233	68.73	
No	106	31.27	
**If yes, what type of water source is it?**			**0.635**
Borehole	73	29.2	
Protected Hand dug well	59	23.6	
Unprotected Hand dug well	32	12.8	
Mechanized borehole	81	32.4	
Mechanized hand dug well	5	2	
**How did you obtain the water facility**			**0.024**
Private	206	78.63	
NGO	30	11.45	
Community built	26	9.92	
**How old is the water facility**			**0.007**
<1 year	27	10.38	
1–10 years	176	67.69	
11–20 years	43	16.54	
>20 years	14	5.38	
**Do you drink this source of water**			**0.147**
Yes	149	51.74	
No	139	48.26	
**What is the main source of water for members of your household**			< **0.001**
Piped water home	56	16.62	
Piped water to yard	16	4.75	
Public standpipe	48	14.24	
Borehole	81	24.04	
Protected dug well	62	18.4	
Unprotected dug well	64	18.99	
Other (specify)	10	2.97	

The large number of residents in our study who are not connected to the GWCL piped systems would imply that many would have to rely on their own initiative to obtain water for domestic purposes. In their study [46], discovered a large number of private or self-supplied water sources delivery in Ghana's urban areas. Furthermore [7], discovered that self-supply water has evolved into a water-supply strategy for residents in urban and peri-urban areas. According to Ref. [47], where groundwater is easily accessible, deprived municipal homes, in particular, may opt for self-supply. Self-supply of groundwater, on the other hand, has implications for drinking water supplies and user health [14].

Moreover, approximately 233 (68.73 %) of respondents had an alternative water source, whereas 106 (31.27 %) did not. Mechanized boreholes accounted for 81 (32.40 %), boreholes for 73 (29.20 %), protected hand dug wells for 59 (23.60 %), unprotected hand dug wells for 32 (12.80 %), and mechanized hand dug wells for 5 (2.00 %) (Table 2). However, there was insignificant association between having an alternative source of water and the type of water source of respondents (p = 0.138) at the 5 % level of significance (Table 2). According to our findings, 69 % of respondents had access to alternative water sources, with mechanized boreholes (32 %) being the most common (Table 2). This finding relates to Ref. [7] who conducted a similar study in Dodowa, Accra, and discovered that approximately 55 % of the residents had access to alternative water sources with boreholes accounting for 38 %. The researchers discovered that residents prefer to meet their own water needs rather than rely on state agencies or private entities. Other studies in Kumasi have revealed that the reliance on alternative sources of water such as hand-dug wells, boreholes, springs, and surface water is due to inconsistencies in water supply or lack of access to supply from Ghana Water Company Limited (GWCL) [24, 29,37]. Our findings support the observation made by Ref. [9], who stated that the increasing reliance on self-supply water could be attributed to rising urban population with increasing water demand and the low cost of drilling a well.

In addition, our findings supports the observation by Ref. [48], that the provision of self-supply water can be attributed to inadequate water supply by state agencies. This finding, however, contrasts with the findings of [24] in Oforikrom, where only 9 % of households obtained additional drinking water from protected hand-dug wells (1 %) and boreholes (8 %).

Studies have shown that people who are not connected to piped water usually resort to alternative water sources to meet their daily water supply [8,14,15]. To fill the water supply gap, most residents in peri-urban areas rely on groundwater self-supply [7]. Though self-supply is usually privately owned, the benefits are usually significant because other members of the community can utilize the water facilities [4]. Self-supply is now widely acknowledged as an important factor in delivering water to families in low- and middle-income countries (LMICs) [4].

When asked about how they obtained the facilities, approximately 203 (78.63 %) of respondents reported receiving the water supply facility through private or personal assistance, 30 (11.45 %) through non-governmental organizations (NGOs) and 26 (9.92 %) through community-built water facilities (Table 2). Similarly, when asked about how old the water facility was, a higher proportion of the wells (77 %) have been in operation for one to ten years. There was significant association between age of the facility and the type/source of water (p = 0.007) (Table 2). The facilities in operation from one to ten (1–10) years contributed 68.44 % to the total association (see Supplementary sheet). Thus, a clear indication that the practice of self-supply has been very recent. This could be due to GWCL's inability to supply water to the rapidly developing urban areas of Kumasi. Furthermore, when asked about drinking alternative water sources, the study discovered that 52 % of respondents drank from an alternative water supply facility (Table 2). But, there was no significant association between respondents who drink from alternative water sources and the type of water source (p = 0.147) at the 5 % level of significance (Table 2). However, there is significant association at the 5 % level between the main water source of the respondents and the type of water source (p < 0.001) with boreholes accounting for the majority (24 %) (Table 2). This highlights the importance that authorities should place on self-supply water, as it plays a significant role in filling the gap in water supply. According to Ref. [49], 32 % of the people in Asia-Pacific used self-supplied water for drinking purposes in 2018. Similarly [50], discovered that boreholes were the main drinking water source for 47 % of respondents in Bekasi City, Indonesia. Improving self-supply is another way to reduce demand on public water utility providers [51]. Private self-supply water has become an essential component of the urban water supply system, but it has been largely ignored for some time [9].

### 3.3 Perception of water quality and treatment measures

According to the findings, sachet water 183 (51.84 %) was the respondents' main source of drinking water, followed by piped water to the dwelling 42 (11.90 %), public tap or pipe 38 (10.76 %), borehole 28 (7.93 %), protected dug well 26 (7.37 %), piped water to the yard or property 16 (4.53 %), bottled water 11 (3.12 %), and unprotected dug well 9 (2.55 %) (Table 3). Chi-square analysis revealed that there is significant association between the main drinking water source and the type of water at the 5 % level of significance (p = 0.005) with sachet water contributing (55.74 %) to this significance (see Supplementary sheet). From the outcomes of the research, approximately half of the interviewees relied on sachet water as their main drinking water source. Some studies in Ghana have shown that sachet water is fast becoming the main source of drinking water in both rural and urban areas. For instance, a survey conducted by Ref. [24] in Oforikrom municipality showed that sachet water accounted for 46 % of residents' drinking water sources. Furthermore, a study in Dodowa found that approximately 90 % of respondents purchased sachet water, while a third purchased bottled water for drinking purpose [47]. There is the general perception by respondents that sachet water is safer as compared to sources from GWCL and self-supply.

**Table 3:** Drinking water, perception of water quality and treatment measures & p-values from chi square analysis.

Question	Frequency	Percent (%)	p-value
What is the main drinking water source for members of your household			0.005
Piped water into home	42	11.9	
Piped water to yard	16	4.53	
Public tap	38	10.76	
Borehole	28	7.93	
Protected hand-dug well	26	7.37	
Unprotected hand-dug well	9	2.55	
Bottled water	11	3.12	
Sachet water	183	51.84	
**Is your water supply adequate throughout the year**			**0.692**
Sufficient throughout the year	281	84.89	
Insufficient throughout the year	42	12.69	
Seasonal during the year	8	2.42	
**Perception of the quality of water you use**			**0.151**
Good	251	73.39	
Acceptable	79	23.1	
Bad	12	3.51	
**Do you add anything to your water to make it safer to drink?**			< **0.001**
Yes	96	35.56	
No	174	64.44	
**What do you do to the water safe to drink?**			< **0.001**
Boil	108	49.09	
chlorinate	68	30.91	
Strain through a cloth	2	0.91	
Use a filter (ceramic, sand, etc)	18	8.18	
Allow to settle	21	9.55	
Other (specify)	3	1.36	

Furthermore, when asked about their water adequacy, majority of respondents reported having adequate water supply throughout the year 281 (84.89 %), insufficient throughout the year 42 (12.69 %) and seasonal during the year 8 (2.42 %) (Table 3). There was insignificant association between respondents having adequate water supply and the type of water source (p = 0.692) at the 5 % level of significance (Table 3). However, about 85.00 % of the respondents indicated they had adequate water supply throughout the year (Table 3). This conclusion is in agreement with a recent survey conducted in Kumasi, which established that 87 % of the household population had adequate access to potable water [52]. Furthermore, when they were probed about their perception of water quality, a greater number of respondents 251 (73.39 %) thought it was good, 79 (23.10 %) thought it was acceptable, and 12 (3.51 %) thought it was poor (Table 3). A greater number 174 (64.44 %) did not treat their water to make it drinkable while 96 (35.56 %) treated their water to make it drinkable (Table 3). Our findings support the Ghana Living Standards Survey (GLSS) [18] findings that the majority of Ghanaian households (57.6 %) do not treat their drinking water. Similarly, a study by Ref. [37] in Kumasi found that about 70 % of the respondents reported that they did not treat their groundwater before drinking. Another study in Kenya revealed that about 75.0 % of households did not treat their water before drinking [53], whilst a study in Pakistan found that about 73.0 % of households do not treat their water [54]. Furthermore, a study in Dodowa discovered that three out of every four residents who self-supply water said the water was safe to drink, and that 80% of those interviewed did not treat their drinking water [47]. In addition, a study in Ethiopia found that only 12 % of residents treat their water in any way to make it potable [4]. The perception that groundwater is of high quality, most people who have private water wells believe the water is safe for consumption [9,37]. A study in Kenya found that households treat their water when they perceive the water is unsafe [53] whilst others studies found that those who perceive their water to be safe do not treat [55,56]. According to Ref. [37], the failure to treat their water before consumption could be due to economic reasons or lack of awareness of the associated risks. Thus, this practise could exacerbate the disease burden in such areas, as consumption of unsafe water is associated with a number of diseases, including, diarrhoea, hepatitis A, cholera, typhoid, polio and dysentery [24]. Researchers have found high levels of nitrates in groundwater which when consumed without treatment can lead to methemoglobinemia [[57], [58], [59]] and other health risks associated with excessive metal intake [57]. Among those who treated their water to make it drinkable, majority 108 (49.09 %) boiled their water followed by 68 (30.91 %) who added bleach/chlorine, 21 (9.55 %) let it stand and settled, 18 (8.18 %) used filters, 2 (0.91 %) used cloth and 3 (1.36 %) did not specify the method used (Table 3). This findings is consistent with a study in Pakistan where 47.60 % of those who treat their water used boiling as the cheapest means [54]. Our study found that there was a significant relationship between what residents did to ensure the safety of their water to drink and type of water source (p < 0.001) (Table 3). The analysis revealed that those who boil their water contributed 50.53 % and those who add bleach or chlorine contributed 30.53 % to the association (see Supplementary sheet). This finding highlight the importance of treating water from the source for drinking purposes.

## 4 Strengths and limitations

The findings of this study were compared with other studies with the understanding that the current data cannot be generalized as the situation in Kumasi and other parts of the country and the world, but rather contribute to the country's scant data on self-supply water. The study serves as a baseline for the government, other state agencies, and researchers in Kumasi and Ghana regarding self-supply.

The study was limited to five (5) communities in Kumasi due to time and logistical constraints and hence the results and interpretation might not reflect the exact situation in Kumasi. The preferred respondents were the household heads and respondents who had lived in the house for a longer period of time, but the majority of the respondents were young and may not have been able to provide comprehensive responses to the questions. Although this study asked households about their perceptions of self-supply water quality, no verifiable water quality measurements were taken.

## 5 Conclusion

The key objectives of this research was to ascertain the level of self-supply in five communities in the Kumasi Metropolis. The study revealed that self-supply is widespread in the five communities because majority of the residents are not connected to the Ghana Water Company Limited piped system. The study showed there was significant association between some household characteristics such as religion, household size, duration of stay and the number of dependents and the type of source of water of residents. The study found that sachet water was the preferred source of drinking water for the majority of the people in the study area, confirming the growing belief that it is safer than other water sources. The study revealed the self-supply water has contributed significantly to meeting the water needs of residents in Kumasi's peri-urban and low-income areas. The water facilities in most of the communities were quite recent especially for the boreholes, indicating that some residents are diversifying into deeper groundwater. This study will contribute to the scant literature on the extent of self-supply and assist policymakers in understanding the existing challenges to self-supply in urban and peri-urban areas, as well as in implementing measures to mitigate negative health effects.

Hence, self-supply is playing a pivotal role meeting the water needs of some communities in the Kumasi Metropolis and if properly managed could contribute significantly to the country's accomplishment of SDG 6.1. For this reason, residents should be educated to disinfect their self-supply sources regularly to minimise health implications of drinking contaminated water.

## 6 Recommendation

The Ghana Standards Authority (GSA), Environmental Protection Agency (EPA), Water Resources Commission (WRC), the Metropolitan Assembly and other state enforcement agencies should intensify education and regulations on self-supply of groundwater in urban and peri-urban communities to ensure accessibility to safe drinking water. In future, researchers should conduct more studies to determine the water quality of self-supply to ensure that residents have access to safely managed water. To broaden the scope of the study and the impact of self-supply groundwater, additional studies should be conducted in other communities, as well as the extent of other beneficiaries who have access to self-supply facilities other than the owners.

## Ethics declarations

This study was reviewed and approved by the Committee on Human Research Publication and Ethics (CHRPE) of Kwame Nkrumah University of Science and Technology, with the approval number: CHRPE/AP/546/22.

## Data availability

The data for this study will be made available on request.

## CRediT authorship contribution statement

**Noel Bakobie:** Writing – review & editing, Writing – original draft, Software, Resources, Methodology, Investigation, Formal analysis, Data curation, Conceptualization. **Helen M.K. Essandoh:** Writing – review & editing, Validation, Supervision, Resources, Conceptualization. **Sampson Oduro-Kwarteng:** Writing – review & editing, Supervision, Resources, Conceptualization. **Emmanuel Kwame Appiah-Adjei:** Writing – review & editing, Supervision, Resources, Conceptualization. **Shaikh Ziauddin Ahammad:** Supervision, Conceptualization. **Sumedha Chakma:** Writing – review & editing, Supervision, Conceptualization.

## Declaration of competing interest

The authors declare that they have no known competing financial interests or personal relationships that could have appeared to influence the work reported in this paper.
