# Supplementary material for: Self-supply groundwater in five communities: Moshie Zongo, Aboabo, Kotei, Ayeduase and Apemso in Kumasi Metropolis, Ghana
Source: Heliyon. 2023 Dec 19;10(1):e23823. doi: 10.1016/j.heliyon.2023.e23823 (PMC10772620; doi:10.1016/j.heliyon.2023.e23823)
Supplement: Multimedia component 1 [file mmc1.docx]

**SURVEY QUESTIONNAIRE**

**Sanitary survey questionnaire to aid in the understanding of contamination of shallow groundwater from onsite sanitation systems in peri-urban areas in Kumasi.**

**Introduction:**

This research is being carried out as part of my PhD thesis. The purpose of this research is to gain knowledge about household water supply and sanitation facilities and related contamination issues. As part of the questions I would like to see your household’s water and sanitation facilities and the general sanitation situation that exist within the water sources and the potential sources of contamination. Your household has been chosen purposively because you own a water facility in your vicinity. This will enable me to have a general view of the sanitation situation and water supply issues. The information given is only for academic purposes and your confidentiality will be assured.

**GENERAL INFORMATION**

Questionnaire No.: …………………………………………………………………….

Name of Interviewer: ………………………………………………………………….

Date of Interview (dd/mm/yy): ……………………………………………………….

Time of Interview: ……………………………………………………………………

Location: ……………………………………………………………………………..

House No.:……………………………………………………………………………

Name of Sub-metro: …………………………………………………………………..

Name of Metro: ………………………………………………………………………..

Name of Region: ……………………………………………………………………..

GPS points: Latitudes: ……………………………. Longitudes: ………………………………

**SECTION A: DEMOGRAPHIC INFORMATION**

1. Name of Respondent ……………………………………………………………………..
2. Sex of Respondent 1. Male [ ] 2. Female [ ].
3. Age of respondent: 1. Less than 26 years [ ] 2. 26-35 years [ ] 3. 36-45 years [ ] 4. 46 - 55years [ ] 5. 56 – 65 years [ ] 6. 66+ [ ].
4. Marital status of respondent: 1. Married [ ] 2. Single [ ] 3. Widow / Widower [ ] 4. Divorced [ ] 5. Separated [ ].
5. Educational Status of respondent: 1. Primary [ ] 2. JHS [ ] 3. Secondary [ ] 4. Post-Secondary [ ] 5. Tertiary [ ] 6. None [ ]
6. Occupation of respondent: 1. Self-employed [ ] 2. Government employee [ ] 3. Private sector [ ]
7. Monthly income of respondent: 1. < GHC 500 [ ] 2. 500 – 1000 [ ] 3. 1001 – 2000 [ ] 4. 2001 – 3000 [ ] 5. > 3000 [ ].
8. Religion of respondent: 1. Christian [ ] 2. Muslim [ ] 3. Traditional [ ] 4. Other (specify) ………………………………………………………………………………
9. Household size of respondent: 1. < 5 [ ] 2. 5 – 9 [ ] 3. 10 – 14 [ ] 4. 15 – 20 [ ] 5. > 20 [ ].
10. Number of dependents of respondent: 1. < 3 [ ] 2. 3 - 6 [ ] 3. 7 - 10 [ ] 4. 11+ [ ].
11. Duration of stay of respondent: 1. < 5 years [ ] 2. 5 – 9 years [ ] 3. 10 – 14 years [ ] 4. 15 – 20 years [ ] 5. > 20 years [ ].
12. Type of home: 1. Private [ ] 2. Compound house [ ]

……………………………………………………………………………………………

**SECTION B**

**WATER SUPPLY ISSUES**

1. Are you connected to Ghana Water Company Limited water supply? Yes [ ] 2. No [ ].
2. Do you have any other source of water in this house? 1. Yes [ ] 2. No [ ].

If No, proceed to **question 18.**

1. If yes, what type of water source is it? 1. Borehole [ ] 2. Protected Hand dug well [ ] 3. Unprotected Hand dug well [ ] 4. Mechanized borehole [ ] 4. Mechanized hand dug well [ ] 5.Other (specify) ………………………
2. How did you obtain the water facility? 1. Private [ ] 2. NGO [ ] 3. Community built [ ]
3. How old is the water facility? 1. < 1 year [ ] 2. 1 – 10 years [ ] 3. 11 - 20 years [ ] 4. > 20 years [ ].
4. Do you drink this source of water? Yes [ ] 2. No [ ].
5. What is the main source of water for members of your household?

Piped water into dwelling [ ] 2. Piped water to yard/plot [ ] 3. Public tap/standpipe [ ] 4. Borehole [ ] 5. Protected dug well [ ] 6. Unprotected dug well [ ] 7. Other (specify) ………………………………

1. What is the main source of drinking water for members of your household?

**Just one response.** Piped water into dwelling [ ] 2. Piped water to yard/plot [ ] 3. Public tap/standpipe [ ] 4. Borehole [ ] 5. Protected dug well [ ] 6. Unprotected dug well [ ] 7. Bottled water [ ] 8. Sachet water [ ] 9. Other (specify) ………………………………

1. What is the frequency of water supply from the main source? 1. 24 hour supply [ ] 2. More than once a day [ ] 3. Once a day [ ] 4. Once in two days [ ] 5. Once in three days [ ] 6. Once a week [ ] 7. Other (specify) ……………………………………………………
2. Is your water supply adequate throughout the year? 1. Sufficient throughout the year [ ] 2. Insufficient throughout the year [ ] 3. Seasonal during the year [ ].
3. What is your perception of the quality of water you use? 1. Good [ ] 2. Acceptable [ ] 3. Bad [ ].
4. **If the answer is bad, please indicate why?** 1. Water is Salty [ ] 2. Water smells [ ] 3. Water is rusty [ ] 4. Others (specify) ……………………………………………………
5. Do you treat your water in any way to make it safer to drink? 1. Yes [ ] 2. No [ ].
6. What do you usually do to the water to make it safer to drink? 1. Boil [ ] 2. Add bleach/chlorine [ ] 3. Strain it through a cloth [ ] 4. Use a filter (ceramic, sand, etc) [ ] 5. Solar disinfection [ ] 6. Let it stand and settle [ ] 7. Other (specify) ……………… …………………………………………………
