# Supplementary material for: Self-supply groundwater in five communities: Moshie Zongo, Aboabo, Kotei, Ayeduase and Apemso in Kumasi Metropolis, Ghana
Source: Heliyon. 2023 Dec 19;10(1):e23823. doi: 10.1016/j.heliyon.2023.e23823 (PMC10772620; doi:10.1016/j.heliyon.2023.e23823)
Supplement: Multimedia component 2 [file mmc2.docx]

**Supplementary Sheet**

Association between respondent characteristics and type of water supply among households.

First row shows frequencies; second row shows the percentage contribution of each category in each respondent characteristic to the total significance of the p-values.

|  | **Mechanized borehole** | **Borehole** | **Protected Hand dug well** | **Unprotected Hand dug well** | **Mechanized hand dug well** | **Total** | **p-Value** |
| --- | --- | --- | --- | --- | --- | --- | --- |
| **Sex** |  |  |  |  |  |  | **0.719** |
| Male | 30 | 25 | 25 | 12 | 1 | 93 |  |
|  | 12.00% | 10.00% | 10.00% | 4.80% | 0.40% | 37.30% |  |
| Female | 48 | 48 | 34 | 21 | 5 | 156 |  |
|  | 19.30% | 19.30% | 13.70% | 8.40% | 2.00% | 62.70% |  |
| **Age** |  |  |  |  |  |  | **0.706** |
| Less than 26 years | 11 | 20 | 9 | 4 | 1 | 45 |  |
|  | 4.40% | 8.00% | 3.60% | 1.60% | 0.40% | 18.10% |  |
| 26 - 35 years | 27 | 22 | 18 | 8 | 2 | 77 |  |
|  | 10.80% | 8.80% | 7.20% | 3.20% | 0.80% | 30.90% |  |
| 36 - 45 years | 23 | 13 | 13 | 8 | 2 | 59 |  |
|  | 9.20% | 5.20% | 5.20% | 3.20% | 0.80% | 23.70% |  |
| 46 - 55 years | 7 | 9 | 8 | 4 | 1 | 29 |  |
|  | 2.80% | 3.60% | 3.20% | 1.60% | 0.40% | 11.60% |  |
| 56 - 65 years | 6 | 4 | 5 | 4 | 0 | 19 |  |
|  | 2.40% | 1.60% | 2.00% | 1.60% | 0.00% | 7.60% |  |
| 66 and above | 4 | 4 | 7 | 5 | 0 | 20 |  |
|  | 1.60% | 1.60% | 2.80% | 2.00% | 0.00% | 8.00% |  |
| **Marital status** |  |  |  |  |  |  | **0.484** |
| Married | 55 | 39 | 43 | 23 | 5 | 165 |  |
|  | 21.91% | 15.54% | 17.13% | 9.16% | 1.99% | 65.74% |  |
| Single | 15 | 26 | 12 | 4 | 1 | 58 |  |
|  | 5.98% | 10.36% | 4.78% | 1.59% | 0.40% | 23.11% |  |
| Widow/widower | 6 | 6 | 4 | 5 | 0 | 21 |  |
|  | 2.39% | 2.39% | 1.59% | 1.99% | 0.00% | 8.37% |  |
| Divorced | 2 | 1 | 1 | 0 | 0 | 4 |  |
|  | 0.80% | 0.40% | 0.40% | 0.00% | 0.00% | 1.59% |  |
| Separated | 1 | 1 | 0 | 1 | 0 | 3 |  |
|  | 0.40% | 0.40% | 0.00% | 0.40% | 0.00% | 1.20% |  |
| **Educational level** |  |  |  |  |  |  | **0.130** |
| Primary | 10 | 12 | 12 | 5 | 1 | 40 |  |
|  | 3.98% | 4.78% | 4.78% | 1.99% | 0.40% | 15.94% |  |
| JHS | 12 | 10 | 21 | 8 | 2 | 53 |  |
|  | 4.78% | 3.98% | 8.37% | 3.19% | 0.80% | 21.12% |  |
| Secondary | 22 | 19 | 2 | 6 | 1 | 50 |  |
|  | 8.76% | 7.57% | 0.80% | 2.39% | 0.40% | 19.92% |  |
| Post-Secondary | 8 | 5 | 6 | 5 | 1 | 25 |  |
|  | 3.19% | 1.99% | 2.39% | 1.99% | 0.40% | 9.96% |  |
| Tertiary | 19 | 21 | 17 | 5 | 1 | 63 |  |
|  | 7.57% | 8.37% | 6.77% | 1.99% | 0.40% | 25.10% |  |
| None | 7 | 6 | 2 | 4 | 0 | 19 |  |
|  | 2.79% | 2.39% | 0.80% | 1.59% | 0.00% | 7.57% |  |
| Other | 1 | 0 | 0 | 0 | 0 | 1 |  |
|  | 0.40% | 0.00% | 0.00% | 0.00% | 0.00% | 0.40% |  |
| Occupation |  |  |  |  |  |  | **0.310** |
| Self-employed | 20 | 25 | 19 | 13 | 3 | 80 |  |
|  | 8.00% | 10.00% | 7.60% | 5.20% | 1.20% | 32.00% |  |
| Trader | 23 | 22 | 14 | 10 | 1 | 70 |  |
|  | 9.20% | 8.80% | 5.60% | 4.00% | 0.40% | 28.00% |  |
| Government employee | 15 | 6 | 3 | 4 | 1 | 29 |  |
|  | 6.00% | 2.40% | 1.20% | 1.60% | 0.40% | 11.60% |  |
| Private sector | 4 | 3 | 4 | 0 | 0 | 11 |  |
|  | 1.60% | 1.20% | 1.60% | 0.00% | 0.00% | 4.40% |  |
| NGO | 3 | 0 | 0 | 1 | 0 | 4 |  |
|  | 1.20% | 0.00% | 0.00% | 0.40% | 0.00% | 1.60% |  |
| Other | 14 | 16 | 20 | 5 | 1 | 56 |  |
|  | 5.60% | 6.40% | 8.00% | 2.00% | 0.40% | 22.40% |  |
| **Monthly Income** |  |  |  |  |  |  | **0.139** |
| < GHC 500 | 18 | 30 | 22 | 10 | 3 | 83 |  |
|  | 8.53% | 14.22% | 10.43% | 4.74% | 1.42% | 39.34% |  |
| 500 – 1000 | 29 | 27 | 18 | 14 | 2 | 90 |  |
|  | 13.74% | 12.80% | 8.53% | 6.64% | 0.95% | 42.65% |  |
| 1001 – 2000 | 17 | 6 | 5 | 4 | 1 | 33 |  |
|  | 8.06% | 2.84% | 2.37% | 1.90% | 0.47% | 15.64% |  |
| 2001 – 3000 | 4 | 1 | 0 | 0 | 0 | 5 |  |
|  | 1.90% | 0.47% | 0.00% | 0.00% | 0.00% | 2.37% |  |
| Religion |  |  |  |  |  |  | **< 0.001** |
| Christian | 61 | 54 | 22 | 15 | 5 | 157 |  |
|  | 24.50% | 21.69% | 8.84% | 6.02% | 2.01% | 63.05% |  |
| Muslim | 13 | 18 | 35 | 13 | 1 | 80 |  |
|  | 5.22% | 7.23% | 14.06% | 5.22% | 0.40% | 32.13% |  |
| Traditional | 3 | 1 | 3 | 5 | 0 | 12 |  |
|  | 1.20% | 0.40% | 1.20% | 2.01% | 0.00% | 4.82% |  |
| **Household size** |  |  |  |  |  |  | **< 0.001** |
| < 5 | 29 | 37 | 12 | 11 | 3 | 92 |  |
|  | 11.79% | 15.04% | 4.88% | 4.47% | 1.22% | 37.40% |  |
| 5 - 9 | 38 | 25 | 23 | 16 | 3 | 105 |  |
|  | 15.45% | 10.16% | 9.35% | 6.50% | 1.22% | 42.68% |  |
| 10 - 14 | 7 | 7 | 9 | 2 | 0 | 25 |  |
|  | 2.85% | 2.85% | 3.66% | 0.81% | 0.00% | 10.16% |  |
| 15 - 20 | 0 | 3 | 5 | 1 | 0 | 9 |  |
|  | 0.00% | 1.22% | 2.03% | 0.41% | 0.00% | 3.66% |  |
| >20 | 3 | 0 | 11 | 1 | 0 | 15 |  |
|  | 1.22% | 0.00% | 4.47% | 0.41% | 0.00% | 6.10% |  |
| **Number of dependents** |  |  |  |  |  |  | **0.026** |
| <3 | 26 | 40 | 16 | 10 | 5 | 97 |  |
|  | 11.11% | 17.09% | 6.84% | 4.27% | 2.14% | 41.45% |  |
| 3 - 6 | 36 | 23 | 30 | 19 | 1 | 109 |  |
|  | 15.38% | 9.83% | 12.82% | 8.12% | 0.43% | 46.58% |  |
| 7 - 10 | 7 | 6 | 8 | 1 | 0 | 22 |  |
|  | 2.99% | 2.56% | 3.42% | 0.43% | 0.00% | 9.40% |  |
| 11+ | 2 | 0 | 3 | 1 | 0 | 6 |  |
|  | 0.85% | 0.00% | 1.28% | 0.43% | 0.00% | 2.56% |  |
| **Duration of stay** |  |  |  |  |  |  | **0.006** |
| < 5 years | 23 | 28 | 15 | 14 | 2 | 82 |  |
|  | 9.58% | 11.67% | 6.25% | 5.83% | 0.83% | 34.17% |  |
| 5 – 9 years | 29 | 19 | 7 | 3 | 1 | 59 |  |
|  | 12.08% | 7.92% | 2.92% | 1.25% | 0.42% | 24.58% |  |
| 10 – 14 years | 13 | 13 | 11 | 3 | 2 | 42 |  |
|  | 5.42% | 5.42% | 4.58% | 1.25% | 0.83% | 17.50% |  |
| 15 – 20 years | 5 | 4 | 7 | 1 | 0 | 17 |  |
|  | 2.08% | 1.67% | 2.92% | 0.42% | 0.00% | 7.08% |  |
| . > 20 years | 6 | 8 | 17 | 8 | 1 | 40 |  |
|  | 2.50% | 3.33% | 7.08% | 3.33% | 0.42% | 16.67% |  |
| **Type of home** |  |  |  |  |  |  | **0.102** |
| Private | 38 | 30 | 15 | 11 | 3 | 97 |  |
|  | 15.26% | 12.05% | 6.02% | 4.42% | 1.20% | 38.96% |  |
| Compound house | 38 | 42 | 44 | 21 | 3 | 148 |  |
|  | 15.26% | 16.87% | 17.67% | 8.43% | 1.20% | 59.44% |  |
| Others | 3 | 0 | 1 | 0 | 0 | 4 |  |
|  | 1.20% | 0.00% | 0.40% | 0.00% | 0.00% | 1.61% |  |
| **Connected to Ghana Water Company Limited water supply** |  |  |  |  |  |  | **0.024** |
| Yes | 7 | 8 | 15 | 3 | 2 | 35 |  |
|  | 2.80% | 3.20% | 6.00% | 1.20% | 0.80% | 14.00% |  |
| No | 72 | 65 | 44 | 30 | 4 | 215 |  |
|  | 28.80% | 26.00% | 17.60% | 12.00% | 1.60% | 86.00% |  |
| **Other source of water in this house** |  |  |  |  |  |  | **0.138** |
| Yes | 77 | 72 | 44 | 30 | 4 | 227 |  |
|  | 32.90% | 30.80% | 18.80% | 12.80% | 1.70% | 97.00% |  |
| No | 2 | 1 | 1 | 2 | 1 | 7 |  |
|  | 0.90% | 0.40% | 0.40% | 0.90% | 0.40% | 3.00% |  |
| **How did you obtain the facility** |  |  |  |  |  |  | **0.024** |
| Private | 70 | 51 | 44 | 21 | 6 | 192 |  |
|  | 28.46% | 20.73% | 17.89% | 8.54% | 2.44% | 78.05% |  |
| NGO | 5 | 5 | 11 | 6 | 0 | 27 |  |
|  | 2.03% | 2.03% | 4.47% | 2.44% | 0.00% | 10.98% |  |
| Community built | 4 | 12 | 3 | 5 | 0 | 24 |  |
|  | 1.63% | 4.88% | 1.22% | 2.03% | 0.00% | 9.76% |  |
| Other (specify) | 0 | 2 | 0 | 1 | 0 | 3 |  |
|  | 0.00% | 0.81% | 0.00% | 0.41% | 0.00% | 1.22% |  |
| **How old is the facility** |  |  |  |  |  |  | **0.007** |
| < 1 year | 3 | 5 | 6 | 6 | 1 | 21 |  |
|  | 1.23% | 2.05% | 2.46% | 2.46% | 0.41% | 8.61% |  |
| 1 – 10 years | 61 | 52 | 31 | 18 | 5 | 167 |  |
|  | 25.00% | 21.31% | 12.70% | 7.38% | 2.05% | 68.44% |  |
| 11 - 20 years | 11 | 11 | 13 | 7 | 0 | 42 |  |
|  | 4.51% | 4.51% | 5.33% | 2.87% | 0.00% | 17.21% |  |
| > 20 years | 3 | 2 | 9 | 0 | 0 | 14 |  |
|  | 1.23% | 0.82% | 3.69% | 0.00% | 0.00% | 5.74% |  |
| **Do you drink this source of water** |  |  |  |  |  |  | **0.147** |
| Yes | 41 | 27 | 31 | 17 | 5 | 121 |  |
|  | 16.73% | 11.02% | 12.65% | 6.94% | 2.04% | 49.39% |  |
| No | 36 | 43 | 28 | 16 | 1 | 124 |  |
|  | 14.69% | 17.55% | 11.43% | 6.53% | 0.41% | 50.61% |  |
| **Main source of water for members of your household** |  |  |  |  |  |  | **< 0.001** |
| Piped water into dwelling | 7 | 9 | 10 | 1 | 3 | 30 |  |
|  | 3.00% | 3.80% | 4.30% | 0.40% | 1.30% | 12.80% |  |
| Piped water to yard/plot | 5 | 1 | 1 | 2 | 0 | 9 |  |
|  | 2.10% | 0.40% | 0.40% | 0.90% | 0.00% | 3.80% |  |
| Public tap/standpipe | 5 | 16 | 3 | 3 | 2 | 29 |  |
|  | 2.10% | 6.80% | 1.30% | 1.30% | 0.90% | 12.30% |  |
| Borehole | 35 | 33 | 2 | 3 | 0 | 73 |  |
|  | 14.90% | 14.00% | 0.90% | 1.30% | 0.00% | 31.10% |  |
| . Protected dug well | 19 | 3 | 22 | 3 | 0 | 47 |  |
|  | 8.10% | 1.30% | 9.40% | 1.30% | 0.00% | 20.00% |  |
| Unprotected dug well | 1 | 4 | 18 | 19 | 0 | 42 |  |
|  | 0.40% | 1.70% | 7.70% | 8.10% | 0.00% | 17.90% |  |
| Other (specify) | 4 | 1 | 0 | 0 | 0 | 5 |  |
|  | 1.70% | 0.40% | 0.00% | 0.00% | 0.00% | 2.10% |  |
| **Main source of drinking water for members of your household** |  |  |  |  |  |  | **0.005** |
| Piped water into dwelling | 6 | 8 | 7 | 2 | 3 | 26 |  |
|  | 2.46% | 3.28% | 2.87% | 0.82% | 1.23% | 10.66% |  |
| Piped water to yard/plot | 0 | 1 | 4 | 3 | 0 | 8 |  |
|  | 0.00% | 0.41% | 1.64% | 1.23% | 0.00% | 3.28% |  |
| Public tap/standpipe | 2 | 12 | 5 | 7 | 2 | 28 |  |
|  | 0.82% | 4.92% | 2.05% | 2.87% | 0.82% | 11.48% |  |
| Borehole | 12 | 6 | 2 | 2 | 0 | 22 |  |
|  | 4.92% | 2.46% | 0.82% | 0.82% | 0.00% | 9.02% |  |
| Protected dug well | 5 | 3 | 4 | 1 | 0 | 13 |  |
|  | 2.05% | 1.23% | 1.64% | 0.41% | 0.00% | 5.33% |  |
| Unprotected dug well | 0 | 1 | 2 | 1 | 0 | 4 |  |
|  | 0.00% | 0.41% | 0.82% | 0.41% | 0.00% | 1.64% |  |
| Bottled water | 3 | 1 | 1 | 0 | 0 | 5 |  |
|  | 1.23% | 0.41% | 0.41% | 0.00% | 0.00% | 2.05% |  |
| Sachet water | 48 | 38 | 34 | 16 | 0 | 136 |  |
|  | 19.67% | 15.57% | 13.93% | 6.56% | 0.00% | 55.74% |  |
| Other (specify) | 2 | 0 | 0 | 0 | 0 | 2 |  |
|  | 0.82% | 0.00% | 0.00% | 0.00% | 0.00% | 0.82% |  |
| **Is your water supply adequate throughout the year** |  |  |  |  |  |  | **0.692** |
| Sufficient throughout the year | 60 | 55 | 43 | 24 | 5 | 187 |  |
|  | 26.70% | 24.40% | 19.10% | 10.70% | 2.20% | 83.10% |  |
| Insufficient throughout the year | 7 | 10 | 10 | 5 | 0 | 32 |  |
|  | 3.10% | 4.40% | 4.40% | 2.20% | 0.00% | 14.20% |  |
| Seasonal during the year | 2 | 1 | 3 | 0 | 0 | 6 |  |
|  | 0.90% | 0.40% | 1.30% | 0.00% | 0.00% | 2.70% |  |
| **Perception of the quality of water you use** |  |  |  |  |  |  | **0.151** |
| Good | 58 | 44 | 46 | 20 | 6 | 174 |  |
|  | 23.87% | 18.11% | 18.93% | 8.23% | 2.47% | 71.60% |  |
| Acceptable | 17 | 25 | 9 | 11 | 0 | 62 |  |
|  | 7.00% | 10.29% | 3.70% | 4.53% | 0.00% | 25.51% |  |
| Bad | 2 | 2 | 1 | 2 | 0 | 7 |  |
|  | 0.82% | 0.82% | 0.41% | 0.82% | 0.00% | 2.88% |  |
| **Do you treat the water in anyway to make it safer to drink?** |  |  |  |  |  |  | **< 0.000** |
| Yes | 33 | 12 | 26 | 10 | 1 | 82 |  |
|  | 15.57% | 5.66% | 12.26% | 4.72% | 0.47% | 38.68% |  |
| No | 35 | 50 | 20 | 20 | 5 | 130 |  |
|  | 16.51% | 23.58% | 9.43% | 9.43% | 2.36% | 61.32% |  |
| **What do you usually do to the water to make it safer to drink** |  |  |  |  |  |  | **< 0.000** |
| Boil | 23 | 39 | 17 | 17 | 0 | 96 |  |
|  | 12.11% | 20.53% | 8.95% | 8.95% | 0.00% | 50.53% |  |
| Add bleach/chlorine | 19 | 7 | 23 | 8 | 1 | 58 |  |
|  | 10.00% | 3.68% | 12.11% | 4.21% | 0.53% | 30.53% |  |
| Strain it through a cloth | 0 | 1 | 0 | 1 | 0 | 2 |  |
|  | 0.00% | 0.53% | 0.00% | 0.53% | 0.00% | 1.05% |  |
| Use a filter (ceramic, sand, etc) | 15 | 0 | 0 | 0 | 0 | 15 |  |
|  | 7.89% | 0.00% | 0.00% | 0.00% | 0.00% | 7.89% |  |
| Let it stand and settle | 3 | 9 | 3 | 2 | 0 | 17 |  |
|  | 1.58% | 4.74% | 1.58% | 1.05% | 0.00% | 8.95% |  |
| Other (specify) | 1 | 0 | 0 | 0 | 1 | 2 |  |
|  | 0.53% | 0.00% | 0.00% | 0.00% | 0.53% | 1.05% |  |

**Chi-Square analysis, Pearson (df-degree of freedom, N = number of cases, chi-square value), Phi value, and Cramer’s values and p-values**

| SN | **Association between respondent characteristics and type of water supply among households.** | **df** | **N** | **chi-square value** | **Phi value** | **Cramer's V value** | **p-value** |
| --- | --- | --- | --- | --- | --- | --- | --- |
| 1 | Sex | 4 | 249 | 2.088 | 0.092 | 0.092 | 0.719 |
| 2 | Age | 20 | 249 | 16.166 | 0.255 | 0.127 | 0.706 |
| 3 | Marital status | 16 | 251 | 15.559 | 0.249 | 0.124 | 0.484 |
| 4 | Educational level | 24 | 251 | 31.875 | 0.358 | 0.178 | 0.130 |
| 5 | Occupation | 20 | 250 | 22.578 | 0.301 | 0.150 | 0.310 |
| 6 | Monthly Income | 12 | 211 | 17.282 | 0.286 | 0.165 | 0.139 |
| 7 | Religion | 8 | 249 | 43.403 | 0.418 | 0.295 | < 0.001 |
| 8 | Household size | 16 | 246 | 41.421 | 0.410 | 0.205 | < 0.001 |
| 9 | Number of dependents | 12 | 234 | 23.264 | 0.315 | 0.182 | 0.026 |
| 10 | Duration of stay | 16 | 240 | 33.449 | 0.373 | 0.187 | 0.006 |
| 11 | Type of home | 8 | 249 | 13.289 | 0.231 | 0.163 | 0.102 |
| 12 | Are you connected to Ghana Water Company Limited water supply | 4 | 250 | 11.212 | 0.212 | 0.212 | 0.024 |
| 13 | Other source of water in this house | 4 | 234 | 6.966 | 0.173 | 0.173 | 0.138 |
| 14 | How did you obtain the facility | 12 | 246 | 23.427 | 0.309 | 0.178 | 0.024 |
| 15 | How old is the facility | 12 | 244 | 27.119 | 0.333 | 0.192 | 0.007 |
| 16 | Do you drink this source of water | 4 | 245 | 6.796 | 0.167 | 0.167 | 0.147 |
| 17 | Main source of drinking water for members of your household | 24 | 235 | 148.441 | 0.795 | 0.397 | < 0.001 |
| 18 | What is the main source of drinking water for members of your household | 32 | 244 | 56.696 | 0.482 | 0.241 | 0.005 |
| 19 | Is your water supply adequate throughout the year | 8 | 225 | 5.598 | 0.158 | 0.112 | 0.692 |
| 20 | Perception of the quality of water you use | 8 | 243 | 12.009 | 0.222 | 0.157 | 0.151 |
| 21 | Do you treat the water in any way to make it safer to drink? | 4 | 212 | 20.305 | 0.309 | 0.309 | < 0.001 |
| 22 | What do you usually do to the water to make it safer to drink | 20 | 190 | 108.947 | 0.757 | 0.379 | < 0.001 |
